# Supplementary material for: FastqPuri: high-performance preprocessing of RNA-seq data
Source: BMC Bioinformatics. 2019 May 3;20:226. doi: 10.1186/s12859-019-2799-0 (PMC6500068; doi:10.1186/s12859-019-2799-0)
Supplement: Supplementary file 2 — Archive of FastqPuri. Archive containing all files needed to install and run FastqPuri v1.0.6. Date stamp March 22, 2019. (GZ 47,819 kb) [file 12859_2019_2799_MOESM2_ESM.gz › FastqPuri-1.0.6/html/struct__iparam__makeTree.html]

FastqPuri: \_iparam\_makeTree Struct Reference


FastqPuri

Public Attributes |
List of all members

\_iparam\_makeTree Struct Reference

contains makeTree input parameters
More...

`#include <init_makeTree.h>`

|  |  |
| --- | --- |
| Public Attributes | |
| char \* | inputfasta |
|  | |
| char | outputfile [MAX\_FILENAME] |
|  | |
| int | L |
|  | |

## Detailed Description

contains makeTree input parameters

## Member Data Documentation

## ◆ inputfasta

char\* \_iparam\_makeTree::inputfasta

fasta input file

## ◆ L

int \_iparam\_makeTree::L

tree depth

## ◆ outputfile

char \_iparam\_makeTree::outputfile[MAX\_FILENAME]

outputfile path

---

The documentation for this struct was generated from the following file:

- include/init\_makeTree.h


---

Generated on Mon Mar 19 2018 23:42:01 for FastqPuri by  

 1.8.14
